# MicroRNAs as potential biomarkers and therapeutic targets in age-related macular degeneration

**DOI:** 10.3389/fopht.2023.1023782

**Published:** 2023-02-15

**Authors:** Marisa Cruz-Aguilar, Sergio Groman-Lupa, María C. Jiménez-Martínez

**Affiliations:** ^1^ Department of Immunology and Research Unit, Institute of Ophthalmology “Conde de Valenciana Foundation”, Ciudad de México, Mexico; ^2^ Retina Service, Codet Vision Institute, Tijuana, Baja California, Mexico; ^3^ Department of Biochemistry, Faculty of Medicine, National Autonomous University of Mexico, Ciudad de México, Mexico

**Keywords:** biomarkers, age-related macular degeneration, microRNAs, neovascularization, oxidative stress, inflammation, therapeutic targets

## Abstract

Age-related macular degeneration (AMD) involves degenerative and neovascular alteration in the macular region of the retina resulting in central vision loss. AMD can be classified into dry (dAMD) and wet AMD (wAMD). There is no established treatment for dAMD, and therapies available for wAMD have limited success. Diagnosis in early AMD stages is difficult due to the absence of clinical symptoms. Currently, imaging tests are used in the diagnosis of AMD, but cannot predict the clinical course. The clinical limitations to establishing a diagnosis of AMD have led to exploration for innovative and more sensitive tests to support the diagnosis and prognosis of the disease. MicroRNAs (miRNAs) are small single-stranded non-coding RNA molecules that negatively regulate genes by post-transcriptional gene silencing. Because these molecules are dysregulated in various processes implicated in the pathogenesis of AMD, they could contribute to the early detection of the disease and monitoring of its progression. Studies of miRNA profiling have indicated several miRNAs as potential diagnostic biomarkers of AMD, but no approved biomarker is available at present for early AMD detection. Thus, understanding the function of miRNAs in AMD and their use as potential biomarkers may lead to future advances in diagnosis and treatment. Here we present a brief review of some of the miRNAs involved in regulating pathological processes associated with AMD and discuss several candidate miRNAs proposed as biomarkers or therapeutic targets for AMD.

## 1 Introduction

Age-related macular degeneration (AMD) is the leading cause of central retinal blindness and affects around 200 million older individuals worldwide, a number that is expected to increase in the coming years, given the aging global population (1, 2). AMD manifests as dark spots in the central visual field and an inability to distinguish fine details (3). Currently, the diagnosis of AMD is determined by clinical symptoms, fundus photography, optical coherence tomography (OCT), and fluorescein fundus angiography (4). Multiple risk factors, such as genetics, aging, cigarette smoking, light exposure, and poor nutrition play substantial roles in the pathophysiology of AMD (3–8). The cause of AMD is unknown, but several theories have been proposed and inflammation, oxidative stress, and angiogenesis appear to play important roles in its pathogenesis and progression (9).

**Figure 1:**
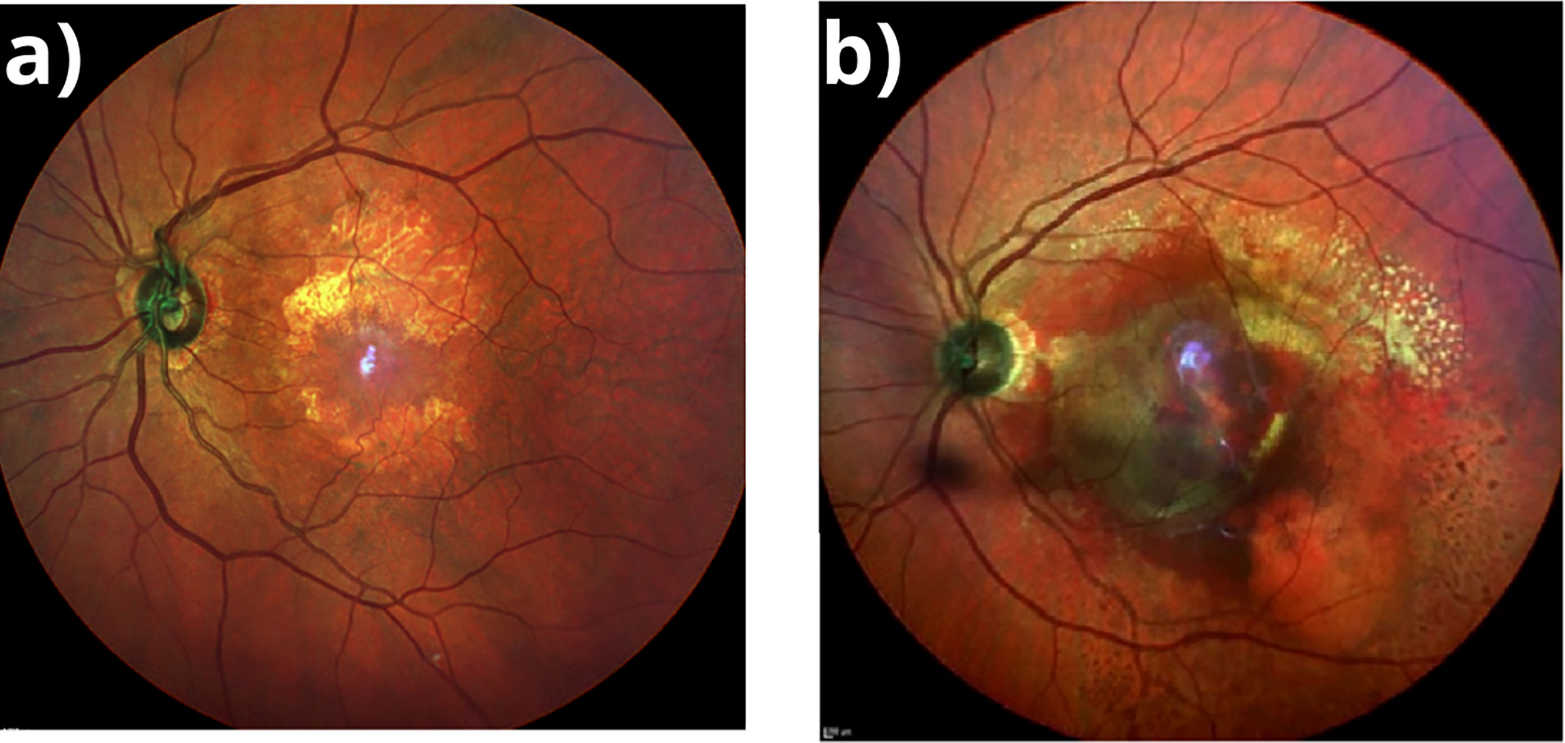
**(A)** Geographic Atrophy. Fundus multicolor photography showing a demarcated area in the macular region, hypopigmented with visible underlying choroidal vessels corresponding to atrophic retina. **(B)** Wet AMD. Fundus multicolor photography showing subretinal hemorrhage involving most of the macular area with exudation and subretinal fluid.

The onset of disease usually is asymptomatic and may be an incidental finding of macular pigmentary changes on dilated fundus examination (10, 11). There are two clinical forms of AMD, dry AMD (dAMD) and wet AMD (wAMD). Early stages of AMD are dry forms of disease, which accounts 80-90% of cases (12). dAMD is associated with yellow deposits called drusen in the space between the retinal pigment epithelium (RPE) and Bruch´s membrane, in addition an altered RPE pigment distribution (3, 11). In late stage dAMD there is geographic atrophy (GA) of the RPE (**Figure 1A**) and degeneration of photoreceptors (11, 12). There is currently no approved or effective treatment to prevent the onset or progression of GA (13, 14). Another variant of advanced AMD is wAMD, responsible for 90% of severe central vision loss. Choroidal neovascularization (CNV) is a key feature of wAMD. The hallmark of CNV (**Figure 1B**) is the growth of new abnormal blood vessels from the choroid into and within the sub-RPE or the subretinal space. The new vessels constitute the choroidal neovascular membrane, which can lead to hemorrhaging and exudation in the retina and profound vision loss (15). Inhibitors of vascular endothelial growth factor (VEGF) are used to treat CNV but with limited success (15–18). The success of therapy largely depends on early diagnosis of dAMD with subsequent regular ophthalmologic examination, which, in addition, facilitates early diagnosis of a conversion to wAMD and timely onset of therapy.

### 1.1 MicroRNAs

MicroRNAs (miRNAs) are small (18–22 nucleotide), single-stranded, noncoding RNAs that negatively regulate gene expression post-transcriptionally (19, 20). These are found in various species, impacting a wide range of cellular and developmental processes (21). They can be isolated from tissues and body fluids such as serum, saliva, and urine, and are remarkably stable (22). A single miRNA may regulate the expression of multiple genes and entire pathways by binding on conserved 3´-untranslated regions (3’-UTR) of messenger RNAs (mRNA) (21).

More than 2500 miRNAs have been described in humans. Hundreds of them are expressed in the retina, where they finely regulate gene expression necessary for visual function (23). Different factors can alter the expression or activity of miRNAs in the retina, affecting cellular processes involved in the pathogenesis of AMD (19, 20, 24).

Various studies have shown panels of miRNAs that have been dysregulated, either in AMD subjects or in AMD experimental models. Such miRNAs have been suggested as potential biomarkers, diagnostic tools, or attractive targets for the control and treatment of this disease. Thus, deregulated expression of a miRNA could be related to an increased risk of developing AMD (25–31).

Dysregulated miRNAs have potential for treatment *via* use of miRNA mimics or anti-miRNAs to modulate cellular function in the retina during AMD (32–34).

This review will briefly describe the current state of knowledge about the influence of the most important miRNAs in the pathogenesis and treatment of AMD, highlighting the need for additional clinical studies to fully understand their importance in ophthalmological practice and to focus on miRNAs as biomarkers and potential therapeutic targets in AMD.

## 2 miRNAs in AMD

**Figure 2:**
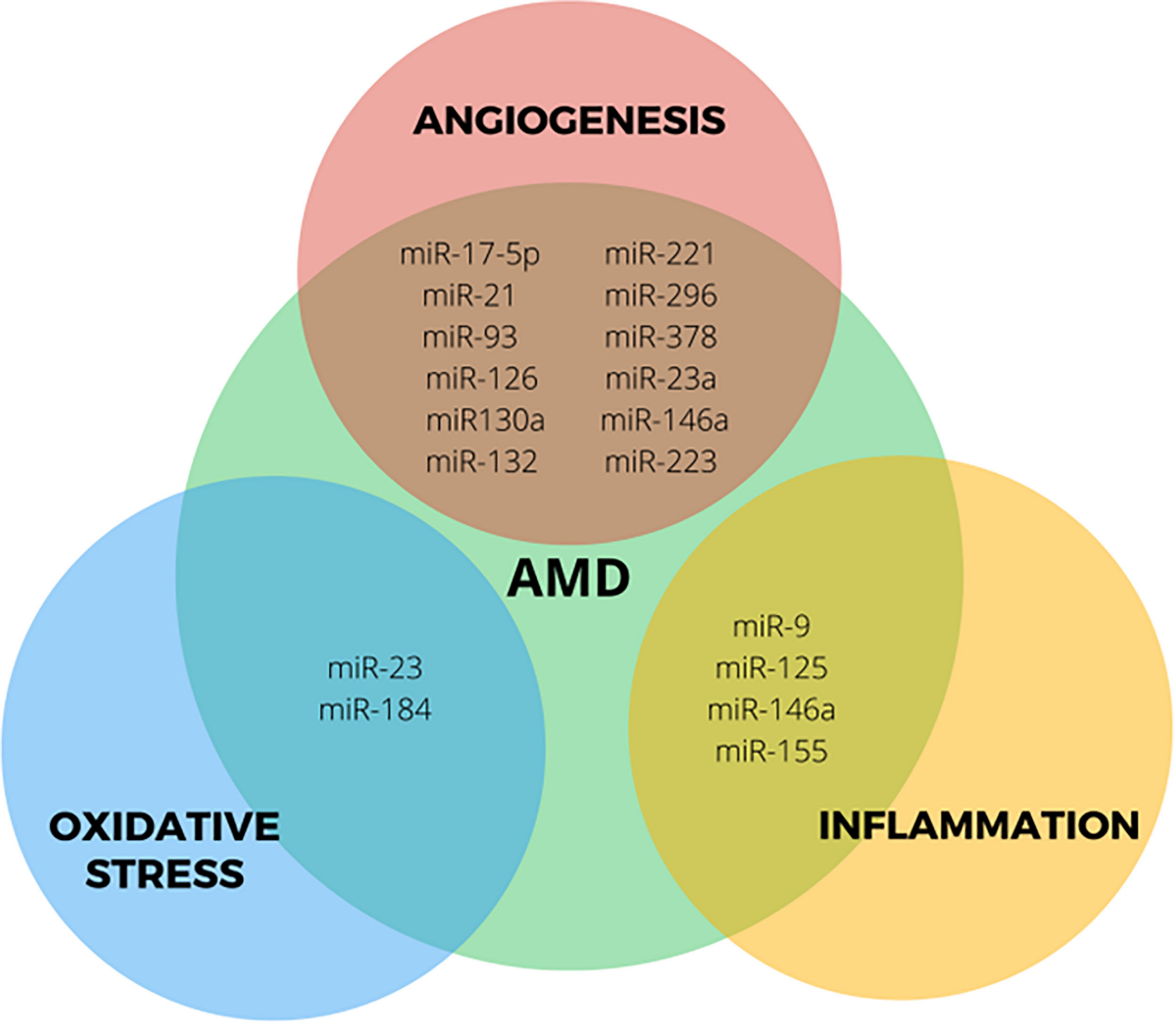
Role of most studied miRNAs deregulated in processes associated with AMD.

The exact etiology of AMD is unknown. Different mechanisms and pathways have been proposed as possibly involved in the pathogenesis of the disease. Therefore, the molecules participating in these pathways are attractive targets for the control and treatment of AMD (24, 32). Different studies have confirmed the aberrant expression of miRNAs in AMD in animals and humans. miRNAs with dysregulated expression are related to alterations in oxidative stress, inflammation, and angiogenesis, processes involved in the pathogenesis of AMD (**Figure 2**) (23, 35). Clinical studies have been undertaken to elucidate miRNAs specific to patients with any type of AMD compared to controls and in different specimens to identify biomarkers of high sensitivity and specificity in the early stages of AMD (35).

Several studies have proposed the detection of dysregulated miRNAs in the early diagnosis of diabetic retinopathy and Alzheimer’s disease (35–37). It is hoped that miRNA profiling will also provide new insights into the pathophysiology of AMD and biomarkers for a more accurate diagnosis and probable prognosis of the course of AMD.

Many dysregulated miRNAs have been identified in AMD. The following sections summarize the work done to profile miRNA dysregulation in AMD patients and prominent miRNAs involved in the most important molecular mechanisms for AMD to identify possible miRNA-based biomarkers.

### 2.1 miRNAs profiles of AMD patients

dAMD often presents asymptomatically. dAMD can progress to wAMD form, resulting in irreversible changes in central vision. Ophthalmological examination cannot always detect retinal damage.

Molecular studies may find a way to diagnose early AMD and gauge retinal damage. One of the first of several clinical studies on miRNA profile analysis in AMD patients was conducted by Ertekin et al., 2014 and focused on the expression profile in wAMD. In that study, miRNAs in plasma from a cohort of 33 wAMD patients and 31 healthy controls (HC) were evaluated using reverse transcription polymerase chain reaction (RT-PCR) and assessed the potential of these miRNAs as diagnostic biomarkers for AMD. In the patient group, 11 miRNAs were significantly downregulated (miR-21, miR-25-3p, miR-140, miR-146b-5p, miR-192, miR-335, miR-342, miR-374a, miR-410, miR-574-3p, and miR-660-5p), 10 miRNAs were specific to the wAMD group (miR-26b-5p, miR-27b-3p, miR-29a-3p, miR-139-3p, miR-212-3p, miR-324-3p, miR-324-5p, miR-532-3p, miR-744-5p, let-7c). Five dysregulated miRNAs targeting the VEGFA gene were upregulated (miR-20b-5p, miR-24-3p, miR-106a-5p, miR-17-5p) and miR-335-5p was downregulated (25).

A next-generation sequencing study of circulating miRNAs in plasma identified 203 dysregulated miRNAs in wAMD patients, but only three of them (miR-301a-3p, miR-361, and miR-424) were significantly downregulated in wAMD patients compared to HC. These three miRNAs regulate pathways involved in angiogenesis, responses to stress and injury, and the regulation of transforming growth factor-beta (26).

The dysregulated miRNAs in a large cohort of 150 wAMD and 150 dAMD patients have also been analyzed (34). Both groups showed higher expression of let-7, miR-301-5p, miR-424-5p, miR-438, miR-661, miR-889, miR-3121 and miR-4258 in serum compared to 200 HC. The expression of miR-661 and miR-3121 was greater in dAMD than wAMD, while that of miR-4258, miR-889, and let-7 was greater in wAMD than dAMD. No significant differences were found between dAMD and wAMD in the levels of miR-301-5p, miR-424-5p, or miR-438 (34). Furthermore, they found a positive correlation between the expression of Let-7 in wAMD patients and the expression of VEGF at both protein and mRNA levels.

To date, only one study has used vitreous humor to profile miRNAs in a small number of patients with wAMD (13 patients) and a control group (13 individuals) using microarrays. The results showed an increase in the levels of miR-146a-5p and miR-548a but a decrease in miR-106b, miR-152, and miR-205. Interestingly, they profiled the levels of miR-106b, miR-146a, and miR-152 in plasma, and these profiles mirrored the findings in the vitreous humor. These results suggest that certain miRNAs are specific to wAMD, and possibly allow monitoring disease progression to wAMD. In addition, a study using the vitreous humor and receiver operating characteristic curve analysis has shown that the area under the curve for miR-146a/miR-106b is high, at 0.97, indicating its potential to distinguish between wAMD and controls (27).

In one study, blood samples of both dry and wet AMD patients have been used. In both groups of patients, expression of miR-27a-3p, miR-29b-3p, and miR-195-5p was significantly increased. Since miR-27a-3p was significantly higher in the wet AMD group, it may be an indicator of choroidal neovascularization formation. Although changes in the expression of miRNAs were found in the blood samples, this may not be reflected locally during the course of AMD (28).

Pogue et al. using miRNA array analysis of 17 AMD whole retinal tissue samples at the macular region and 10 control retinal tissue samples, found that expression of inflammatory miRNAs such as miR-125b, miR-146a, and miR-155 differed between AMD and control tissue (29). These miRNAs are mainly associated with neuro-inflammatory and/or neurodegenerative processes (30).

Recently, Litwińska et al. analyzed the profile of miRNAs in peripheral blood mononuclear cells (PBMCs) isolated from 175 dAMD, 179 wAMD, and 121 HC subjects. They showed that in PBMCs of wAMD patients, the expression of miR-23a-3p, miR-30b, miR-191-5p, and miR-223-3p was upregulated compared to HC. In dAMD patients, miR-23a-3p, miR-126-3p, miR-126-5p, miR-146a expression was upregulated and miR-16-5p, miR-17-3p, and miR-17-5p expression was downregulated compared to controls. Within the subtypes of AMD, six miRNAs were differentially expressed in PBMCs: two were upregulated in wAMD patients (miR-30b, miR-191-5p), and four were upregulated in dAMD patients (miR-126-3p, miR-126-5p, miR-150-5p, miR-155-5p). Correlations between visual acuity and expression of miRNAs were described. Expression of miR-126-3p, miR-126-5p, and miR-155-5p was positively correlated with visual acuity, whereas miR-191-5p expression was negatively correlated. Such correlations were absent in the control group (31).

Plasma samples from 175 patients with dAMD and 179 with wAMD were compared to those from 121 HC subjects using RT-PCR. The expression of miR-16-5p, miR-17-3p, miR-17-5p, miR-23a-3p, miR-126-5p, miR-146a, and miR-223-3p was upregulated, while miR-21-3p, miR-155-5p, miR-191-5p were decreased in both AMD groups compared with controls. In addition, wAMD patients had significantly higher expression of miR-16-5p, miR-30b, miR-191-5p, and significantly lower expression of miR-23a-3p, than dAMD patients. A negative correlation was reported between visual acuity and blood plasma miR-23a-3p in AMD patients but not in the control group. The expression of these miRNAs was positively correlated with levels of angiogenesis-regulating factors in AMD patients but not in the controls (38).

**Table 1** provides a summary of the miRNAs most frequently detected in the above-mentioned studies.

**Table 1:** Summary of identified miRNAs with dysregulated expression in AMD patients.

Author (year)	Sample	Comparison groups	miRNAs with dysregulated expression
Ertekin et al. (25)	Plasma	wAMD vs control	↑miR-20a-5p, -106a-5p, -24-3p, -17-5p, -223-3p
			↓miR-140-3p, -21-5p, -25-3p, -146b-5p, -192-5p, -335-5p, -342-3p, -374a-5p, -410, -660-5p, -574-3p
Grassmann et al. (26)	Serum	wAMD vs control	↓miR-301a-3p, -361-5p, -424-5p
		wAMD vs dAMD	↓miR-424-5p
Szemraj et al. (34)	Serum	dAMD vs control	↑miR-661, -3121, -4258, -889, -438, -424-5p, -301-5p, let-7
			↑miR-661, -3121, -4258, -889, -438, -424-5p, -301-5p, let-7
		wAMD vs control	↑miR-661, -3121, -4258, -889, -438, -424-5p, -301-5p, let-7
		dAMD vs wAMD	↑ miR-4258, -889, let-7
			↓miR-661, -3121
Ménard et al. (27)	Vitreous humour	wAMD vs control	↑miR-146a
			↓miR-106b, -152
Ménard et al. (27)	Plasma	wAMD vs control	↑miR-146a
			↓miR-106b, -152
Ren et al. (28)	Blood	dAMD vs control	↑ miR-27a-3p, miR-29b-3p, miR-195-5p
		wAMD vs control	↑ miR-27a-3p, miR-29b-3p, miR-195-5p
		dAMD vs wAMD	↑ miR-27a-3p
Pogue and Lukiw (29)	Retinal tissues	AMD vs control	↑miR-125b, -146a, -155
Litwińska et al. (31)	PBMCs	dAMD vs control	↑ miR-23a-3p, -126-3p, -126-5p, -146a, -191-5p
			↓ miR-16-5p, -17-3p, -17-5p
		wAMD vs control	↑ miR-23a-3p, -30b, -191-5p
			↓ miR-16-5p, -17-3p, -150-5p, -155-5p
		wAMD vs dAMD	↑miR-30b, -191-5p
			↓miR-126-3p, -126-5p, -150-5p, -155-5p
Ulańczyk et al. (38)	Plasma	dAMD vs control	↑miR-23a-3p, -126-5p, -16-5p, 17-3p, -17-5p, -223-3p, -93
			↓miR-21-3p, -155-5p
		wAMD vs dAMD	↑miR-16-5p, -30b, -191-5p
			↓miR-23a-3p

PBMCs, peripheral blood mononuclear cells. ↑ upregulation↓ downregulation.

A very large number of dysregulated miRNAs are common to separate studies. This may be due to the limitations of each study, such as cohort size, patient selection criteria, type of specimen analyzed, molecular methodology used for identifying miRNAs, and other factors. These variables make it difficult to identify a unique miRNA or to identify a SMALL group of them with potential as biomarkers of AMD. However, because miR-27a, miR-146a, miR-23a, miR-34a, miR-126, and miR-155 were the most frequently identified miRNAs in these studies, it has been suggested they have potential as biomarkers in AMD. However, it remains necessary to determine the sensitivity and specificity of miRNA-based tests for the detection and prognosis of AMD. It is worth mentioning that these studies only provide us with insights concerning overall miRNA changes in AMD patients.

### 2.2 miRNAs and inflammation

One of the immune responses in AMD patients is elicited by the uncontrolled formation of drusen deposited beneath the RPE, which contains complement and other inflammatory components (1). Disruption of RPE/Bruch´s membrane integrity activates the complement system and chronic inflammation that characterizes AMD (1–3). Mutations or polymorphisms in genes coding for the alternative complement pathway regulators, such as factor H (CFH), are associated with AMD (39, 40). It has been found that the expression of CFH is controlled by several miRNAs, among which miR-9, mir-125, miR-146a, and miR-155 have been most studied. Altered upregulation of these miRNAs consistently inhibits CFH activity, promoting the chronic inflammation that characterizes AMD. This has been shown by several clinical studies in which the expression profiles of miRNAs were compared in whole retinal samples from patients with AMD and healthy controls (41, 42).

Functionally, miR-9 plays an essential role in RPE function. It is regulated by reactive oxygen species, retinoic acid, and proinflammatory cytokines such as tumor necrosis factor-alpha and interleukin-1 (IL-1), which are abundantly expressed in the retina under pathological conditions (43). Different studies carried out on ARPE-19 cells, monocytes, and neutrophils or microglial cells have reported the increased expression of miR-9 when exposed to derivatives of retinoic acid or lipopolysaccharide, then linking this microRNA to the innate immune response (43–46).

Various studies have implicated miR-155 in RPE cell preservation and modulation of their response to inflammatory stimuli, as well as in the regulation of the adaptive immune response and immune cell growth (47, 48). Among the targets identified for miR-155 are IL-6 and IL-1β. In clinical studies conducted in patients with uveitis, it was found that the expression of miR-155 is decreased in both blood mononuclear and dendritic cells compared to healthy patients (49). However, as miR-155 regulates multiple targets in a cell type-specific manner, the mechanism of action is unclear.

miR-146a modulates innate immune responses, inflammation, and the microglial activation state, although it is not yet clear whether the induction of miR-146a is protective or detrimental to the cell. It is upregulated by reactive oxygen species, pro-inflammatory cytokines, and amyloid peptides.

Among the main targets identified for mir-146a are CFH and IL-6, and all are involved in the inflammatory immune response. In patients with dAMD or wAMD, miR-146a is upregulated, resulting in the downregulation of CFH and loss of repression of the inflammatory response, thus promoting the pathogenesis of AMD (41). IL-6, which acts as a pro-inflammatory cytokine associated with the progression of dAMD toward wAMD, is downregulated by miR-146a (27) and in patients with wAMD miR-146 upregulation is negatively correlated with IL-6. This effect was not observed in patients with dAMD (27, 50). Different findings between studies may reflect varied inclusion criteria between clinical studies, in addition to the fact that the mechanism of gene regulation of miR-146 is not fully understood in AMD.

### 2.3 miRNAs in oxidative stress

Oxidative stress has been implicated in the pathogenesis of AMD. The retina has high oxygen metabolism, continual exposure to light, and high concentrations of polyunsaturated fatty acids, so is constantly exposed to oxidative damage generated by reactive oxygen species (51). Several studies have shown that miRNA regulation is involved in cytoprotection in response to oxidative stress (52). miR-23 and miR-184 have been most frequently implicated in genetic regulation and modulation of RPE cell survival in response to oxidative damage (52–54).

miR-23a has been found to be downregulated in human RPE cells from AMD patients. Under normal conditions, it has been observed that miR-23 has a protective role in RPE cells in the presence of oxidative stress (52). In macular RPE cells from donor eyes of AMD patients cultured under different concentrations of H_2_O_2_, the expression of miR-23a was upregulated with reduced cell death at low concentrations of H_2_O_2,_ but its protective effect was abolished when the oxidative stress was significantly increased, and its expression was downregulated (52). A miR-23a binding site has been identified in the 3´-UTR of the Fas gene demonstrating the ability of miR-23a to regulate Fas expression in ARPE-19 cells. These results are consistent with the increased expression of Fas and FasL in photoreceptor monolayers and RPE of neovascular membranes from AMD patients (55). Thus, mir-23a has been suggested as a potential therapeutic target in AMD (55, 56).

RPE cells perform various functions that maintain retinal homeostasis, including disposal of photoreceptors outer segments and retaining recyclable material (52, 54). Therefore, disturbances in RPE function due to dysregulated expression of miRNAs, such as miR-184, may lead to the pathophysiology of AMD.

Studies performed on RPE cell cultures from AMD patients have suggested a potential role for miR-184 in the regulation of phagocytosis. The downregulated expression of miR-184 is correlated with an increase in the level of ezrin, a protein important for actin assembly and regulation of phagolysosomal fusion (57), and a significant reduction of lysosomal-associated membrane protein 1 (LAMP-1). In RPE cells, the interaction between these two proteins is required to form phagocytic vacuoles and is critical to the phagocytic digestion of the external segments of the photoreceptors (58). As a result, the outer segments of unrecycled photoreceptors accumulate between the RPE and Bruch’s membrane, favoring the formation of drusen and promoting the pathophysiology of AMD.

### 2.4 miRNAs in angiogenesis

Pathological angiogenesis in the choroid is part of wAMD. Neovascularization in the choroid can be induced by inflammatory and apoptotic RPE cells. Both *in vitro* and *in vivo* studies have revealed that a number of miRNAs are associated with angiogenesis and CNV mechanisms of wAMD (59, 60). It was found that numerous miRNAs known as “angiomirs” such as miR-17-5p, miR-21, miR-93, miR-126, miR-130a, miR-132, miR-221, miR-296, miR-378, among others, are involved in ocular angiogenic processes and most of these miRNAs target genes related to angiogenesis such as VEGF or insulin-like growth factor 2 (IGF-2) (61).

Recent clinical studies in patients with wAMD showed that dysregulated expression of some miRNAs significantly correlated with angiogenesis regulatory factors such as angiogenin and endostatin. miR-17-5p is upregulated in the blood plasma of AMD patients and is positively correlated with these factors, while upregulated mIR-23a-3p, miR-146a, and miR-223-3p showed a negative correlation (38).

Among the most studied miRNAs, miR-21 was downregulated in plasma from patients with wAMD compared to controls (25). In mice, overexpression of miR-21 reduced laser-induced CNV, suggesting an inhibitory function on neovascularization (62).

miR-93 is predicted to target the 3`-UTR of VEGF-A and is implicated in angiogenesis with controversial roles. Some studies have reported that miR-93 promotes angiogenesis and tumor growth, while others using both *in vivo* and *in vitro* models have found that it works by inhibiting neovascularization and angiogenesis (63, 64).

miR-126 is abundantly expressed on endothelial cells. In *in vitro* assays, its downregulation has been found to inhibit different endothelial cell processes such as cell migration and cell survival (65). Supporting these results, miR-126 knock-out mice is lethal, indicating a crucial role of this miRNA in developmental angiogenesis (32).

These miRNAs are related to the angiogenic process. However, more systematic studies are needed to fully elucidate the molecular mechanisms through which this process is regulated by miRNAs. Since these miRNAs may have multiple target mRNAs in the angiogenic pathways, they may serve as possible therapeutic solution for wAMD.

## 3 miRNAs as potential therapeutic targets

Many studies have shown that several miRNAs are involved in angiogenesis and inflammation, indicating that they may contribute to AMD pathogenesis and are possible therapeutic targets for AMD (30). Romano et al. suggested that miR-9, miR-23a, miR-27a, miR-34a, miR-146a, which were upregulated in AMD, and miR-155, which was downregulated, should be investigated as potential therapeutic targets. MiR-27a, miR-146a, and miR-155 are reportedly associated with inflammatory pathways such as tumor necrosis factor, nuclear factor-kappaB, and hypoxia inducible factor signaling (66).

Research in AMD is principally aimed at finding effective therapies to counteract the progression of dAMD to wAMD. While intravitreal injections with anti-VEGF agents are currently available for wAMD, they elicit a limited response in some patients. It has been suggested that the monthly application of intravitreal injection results in accumulation of the drug in most patients and that while this affects vision it results in a significant economic burden for both the patient and the health system. Therefore, better treatments for AMD with longer-lasting effects are needed. This goal could be achieved through gene therapy (23). In retinal dystrophies such as Leber congenital amaurosis, gene therapy has been shown to be safe and effective, and therefore its application as a treatment for other diseases causing blindness, such as AMD, has been considered (67, 68). Gene therapy uses viral vectors containing the genetic products that are transferred to target cells, using their biological capacities to enter the cell and deposit the genetic material. Given that miRNAs regulate the expression of genes involved in pathogenic processes associated with AMD and that a single miRNA can regulate several pathways, these small molecules are interesting targets for gene therapy to treat AMD. As discussed above, dysregulation in the expression of some miRNAs affects the behavior of their target mRNAs and, consequently, the development of AMD-associated clinical manifestations. By delivering specific endogenous miRNA in appropriate amounts, pathological changes in the retina could be reversed. This is possible by designing a viral vector containing the coding sequence for the miRNA of choice under the direction of a tissue-specific promoter. A single viral vector can harbor multiple miRNAs that can regulate multiple pathways or may degrade or inhibit translation of a single mRNA (23). miRNA-based therapies involve the modulation of pathogenic pathways through antagonists and mimics, so-called antagomiRs and agomiRs, respectively (59). AntagomiRs are chemically engineered oligonucleotides that effectively and specifically silence endogenous miRNAs. AgomiRs are synthetic miRNAs that function as the corresponding natural miRNAs (69). An example of these was described in the CNV mouse model using short hairpin RNAs (shRNAs) targeting VEGF (23). ShRNAs are artificial small RNA molecules that mimic pri- or pre-miRNAs. Upon release and expression in cells, they are processed and function similarly to endogenous miRNAs. In mice treated with a viral vector encoding shRNAs, CNV may be reduced (23). Similarly, several promising miRNAs could be evaluated for future treatment of wet and dry AMD. For example, overexpression of miR-21, miR-31, or miR-150, or silencing miR-23/27 are potential approaches to the treatment of CNV in wAMD (60, 62, 70). However, the efficacy and safety profiles of these miRNAs need to be rigorously tested using animal models.

Conversely, in the same laser-induced CNV mouse model, intravitreal injections of miR-142-3p inhibitor reduced blood vessel density by 46% and decreased microglia area by 30%. The ability of miR-142-3p to inhibit neovascularization and inflammation suggests that it may be a candidate therapeutic target in AMD (71).

The overexpression of miRNAs can also be regulated by introducing miRNA sponges (72). These are non-coding RNA molecules that competitively sequester miRNAs from their endogenous target. Sponge miRNAs can be designed as dual targeting hairpins to simultaneously suppress two or more miRNAs, making them ideal when the goal is to hijack multiple different miRNAs (73, 74).

miRNA-based therapies seem to be promising targets, but the following limitations need to be considered in their evaluation: a) AMD is a multifactorial disease, which complicates the identification of a single miRNA as a therapeutic target, b) targeting an individual miRNA can produce changes in the expression of various genes and c) possible adverse effects of miRNA therapy should be evaluated (69).

Thus, miRNA-based therapy will have to overcome three main problems: identification of the target miRNAs, delivery method, and specificity (65, 69).

Some miRNA-based therapies have undergone early stages of testing, but none have passed phase I/II clinical trials, so more studies and clinical evaluations are needed before their implementation in clinical practice (65).

## 4 Conclusion

Different genetic and environmental targets have been studied as AMD risk factors and potential biomarkers. However, *in vitro* findings have limited application in clinical practice. Dysregulated miRNAs have been observed in various critical processes in AMD pathogenesis. Studies on the profile expression of miRNAs with case-control comparisons have allowed detection of associations between dysregulated miRNAs and AMD, but do not imply causality. Due to their involvement in the central processes of AMD, miRNAs have been suggested as ideal therapeutic treatments and candidates for diagnosis. However, it has been challenging to identify just one or a small group of them, particularly because the individual studies differ in their inclusion criteria, sample types and sizes, and methodology by which miRNAs are identified.

A few studies have tried to identify the specific panel of miRNAs that are differentially expressed in AMD patients. A single study has profiled miRNAs in the vitreous humor, but with a small sample size because samples of diseased eyes or the vitreous humor of patients are difficult to obtain, and detecting miRNAs in serum samples has only generated preliminary, discrepant results. Therefore, further investigation in this area is needed to help identify circulating microRNA biomarkers for both forms of AMD.

Unfortunately, several miRNAs investigated using animal models that mimic AMD do not mirror the miRNAs differentially regulated in AMD patients, so their role in the disease remains unknown.

Further studies are necessary to understand their influence on AMD pathogenesis, to develop feasible methods of application in clinical practice, and to develop future therapies. Although OCT and fluorescein angiography are useful in the diagnosis and monitoring of the progression of AMD, any novel, minimally invasive, and a highly sensitive test would help to assess whether any miRNA dysregulation is detectable before clinical signs of AMD appear. So, it is important to estimate the significance of miRNAs as tools to monitor the progression and predict the course of AMD.

Finally, miRNAs may have important diagnostic or therapeutic applications. To allow significant progress in this field, reproducible miRNA profiles must be established to fully understand their importance in clinical practice.

## Author contributions

The authors contributed equally to this work. All authors contributed to the article and approved the submitted version.

## References

[1] Jager RD Mieler WF MJW. Age–related macular degeneration. N Engl J Med (2008) 358(24):2606–17. doi: 10.1056/NEJMra0801537 18550876

[2] Wong WL Su X Li X Cheung CMG Klein R Cheng CY. Global prevalence of age–related macular degeneration and disease burden projection for 2020 and 2040: A systematic review and meta–analysis. Lancet Glob Health (2014) 2(2):e106–16. doi: 10.1016/S2214-109X(13)70145-1 25104651

[3] Pieramici DJ Bressler SB. Age–related macular degeneration and risk factors for the development of choroidal neovascularization in the fellow eye. Curr Opin Ophthalmol (1998) 9(3):38–46. doi: 10.1097/00055735-199806000-00007 10182098

[4] Bourla DH Young TA. Age–related macular degeneration: A practical approach to a challenging disease. J Am Geriatrics Soc (2006) 54(7):1130–5. doi: 10.1111/j.1532-5415.2006.00771.x 16866687

[5] Kiernan DF Hariprasad SM Rusu IM v. MS WF M Jager RD. Epidemiology of the association between anticoagulants and intraocular hemorrhage in patients with neovascular age–related macular degeneration. Retina (2010) 30(10):1573–8. doi: 10.1097/IAE.0b013e3181e2266d 21060269

[6] Seddon JM George S Rosner B. Cigarette smoking, fish consumption, omega–3 fatty acid intake, and associations with age–related macular degeneration: The US twin study of age–related macular degeneration. Arch Ophthalmol (2006) 124(7):995–1001. doi: 10.1001/archopht.124.7.995 16832023

[7] Chakravarthy U Wong TY Fletcher A Piault E Evans C Zlateva G. Clinical risk factors for age–related macular degeneration: A systematic review and meta–analysis. BMC Ophthalmol (2010) 10(1):31. doi: 10.1186/1471-2415-10-31 21144031 PMC3009619

[8] Mitchell P Smith W Wang JJ. Iris color, skin sun sensitivity, and age–related maculopathy: The blue mountains eye study. Ophthalmology. (1998) 105(8):1359–63. doi: 10.1016/S0161-6420(98)98013-7 9709743

[9] Ding X Patel M Chan CC. Molecular pathology of age–related macular degeneration. Prog Retinal Eye Res (2009) 28(1):1–18. doi: 10.1016/j.preteyeres.2008.10.001 PMC2715284 19026761

[10] Ferris FL Fine SL Hyman L. Age–related macular degeneration and blindness due to neovascular maculopathy. Arch Ophthalmol (1984) 102(11):1640–2. doi: 10.1001/archopht.1984.01040031330019 6208888

[11] Ferris FL Wilkinson CP Bird A Chakravarthy U Chew E Csaky K. Clinical classification of age–related macular degeneration. Ophthalmol (2013) 120(4):844–51. doi: 10.1016/j.ophtha.2012.10.036 PMC11551519 23332590

[12] Holz FG Strauss EC Schmitz–Valckenberg S van Lookeren Campagne M. Geographic atrophy: Clinical features and potential therapeutic approaches. Ophthalmology. (2014) 121(5):1079–91. doi: 10.1016/j.ophtha.2013.11.023 24433969

[13] de J PT. Age–related macular degeneration. N Engl J Med (2006) 355(14):1474–85. doi: 10.1056/NEJMra062326 17021323

[14] Snellen ELM Verbeek ALM van den Hoogen GWP Cruysberg JRM Hoyng CB. Neovascular age–related macular degeneration and its relationship to antioxidant intake. Acta Ophthalmol Scand (2002) 80(4):368–71. doi: 10.1034/j.1600-0420.2002.800404.x 12190777

[15] Jager RD Mieler WF MJ. Age–related macular degeneration. N Engl J Med (2008) 358(24):2606–17. doi: 10.1056/NEJMra0801537 18550876

[16] Nowak JZ. Age–related macular degeneration (AMD): pathogenesis and therapy. Pharmacol Rep (2006) 58:353–63. 16845209

[17] Krebs I Glittenberg C Ansari–Shahrezaei S Hagen S Steiner I BS. Non–responders to treatment with antagonists of vascular endotelial growth factor in age–related macular degeneration. Br J Ophthalmol (2013) 97:1443–6. doi: 10.1136/bjophthalmol-2013-303513 23966368

[18] Nischler C Oberkofler H Ortner C Paikl D Riha W Lang N. Complement factor h Y402H gene polymorphism and response to intravitreal bevacizumab in exudative age–related macular degeneration. Acta Ophthalmol (2011) 89(4):e344–9. doi: 10.1111/j.1755-3768.2010.02080.x 21232084

[19] Bartel DP. MicroRNAs: Genomics, biogenesis, mechanism, and function. Cell (2004) 116(2):281–97. doi: 10.1016/s0092-8674(04)00045-5 14744438

[20] Winter J Jung S Keller S Gregory RI Diederichs S. Many roads to maturity: MicroRNA biogenesis pathways and their regulation. Nat Cell Biol (2009) 11(3):228–34. doi: 10.1038/ncb0309-228 19255566

[21] Kim VN. Small RNAs: Classification, biogenesis, and function. Molecules Cells (2005) 19(1):1–15. 15750334

[22] Kosaka N Iguchi H Yoshioka Y Takeshita F Matsuki Y Ochiya T. Secretory mechanisms and intercellular transfer of microRNAs in living cells. J Biol Chem (2010) 285(23):17442–52. doi: 10.1074/jbc.M110.107821 PMC2878508 20353945

[23] Askou AL Alsing S Holmgaard A Bek T Corydon TJ. Dissecting microRNA dysregulation in age–related macular degeneration: new targets for eye gene therapy. Acta Ophthalmologica. (2018) 96(1):9–23. doi: 10.1111/aos.13407 28271607

[24] Ha TY. MicroRNAs in human diseases: From cancer to cardiovascular disease. Immune Netw (2011) 11(3):135–54. doi: 10.4110/in.2011.11.3.135 PMC3153666 21860607

[25] Ertekin S Yildirim Ö Dinç E Ayaz L Balci Fidanci Ş Tamer L. Evaluation of circulating miRNAs in wet age–related macular degeneration. Mol Vis (2014) 20:1057–66. PMC4113960 25221421

[26] Grassmann F Schoenberger PGA Brandl C Schick T Hasler D Meister G. A circulating MicroRNA profile is associated with late– stage neovascular age–related macular degeneration. PLoS One (2014) 9(9):e107461. doi: 10.1371/journal.pone.0107461 25203061 PMC4159338

[27] Ménard C Rezende FA Miloudi K Wilson A Tétreault N Hardy P. MicroRNA signatures in vitreous humour and plasma of patients with exudative AMD. Oncotarget. (2016) 7(15):19171–84. doi: 10.18632/oncotarget.8280 PMC4991373 27015561

[28] Ren C Liu Q Wei Q Cai W He M Du Y. Circulating miRNAs as potential biomarkers of age–related macular degeneration. Cell Physiol Biochem (2017) 41(4):1413–23. doi: 10.1159/000467941 28315863

[29] Pogue AI Lukiw WJ. Up–regulated pro–inflammatory MicroRNAs (miRNAs) in alzheimer’s disease (AD) and age–related macular degeneration (AMD). Cell Mol Neurobiol (2018) 38(5):1021–31. doi: 10.1007/s10571-017-0572-3 PMC11481951 29302837

[30] Bhattacharjee S Zhao Y Dua P Rogaev EI Lukiw WJ. MicroRNA–34α–mediated down–regulation of the microglial–enriched triggering receptor and phagocytosis–sensor TREM2 in age–related macular degeneration. PLoS One (2016) 11(3):e0150211. doi: 10.1371/journal.pone.0150211 26949937 PMC4780721

[31] Litwińska Z Sobuś A Łuczkowska K Grabowicz A Mozolewska–Piotrowska K Safranow K. The interplay between systemic inflammatory factors and MicroRNAs in age–related macular degeneration. Front Aging Neurosci (2019) 11:286. doi: 10.3389/fnagi.2019.00286 31695606 PMC6817913

[32] Zhou Q Anderson C Hanus J Zhao F Ma J Yoshimura A. Strand and cell type–specific function of microRNA–126 in angiogenesis. Mol Ther (2016) 24(10):1823–35. doi: 10.1038/mt.2016.108 PMC5112035 27203443

[33] Creemers EE Tijsen AJ Pinto YM. Circulating MicroRNAs: Novel biomarkers and extracellular communicators in cardiovascular disease? Circ Res (2012) 110:483–95. doi: 10.1161/CIRCRESAHA.111.247452 22302755

[34] Szemraj M Bielecka–Kowalska A Oszajca K Krajewska M Goś R Jurowski P. Serum micrornas as potential biomarkers of AMD. Med Sci Monitor. (2015) 21:2734–42. doi: 10.12659/MSM.893697 PMC4576928 26366973

[35] Martinez B Peplow P. MicroRNAs as diagnostic and prognostic biomarkers of age–related macular degeneration: Advances and limitations. Neural Regeneration Res (2021) 16(3):440–7. doi: 10.4103/1673-5374.293131 PMC7996036 32985463

[36] Lee CS Larson EB Gibbons LE Lee AY McCurry SM Bowen JD. Associations between recent and established ophthalmic conditions and risk of alzheimer’s disease. Alzheimer’s Dementia. (2019) 15(1). doi: 10.1016/j.jalz.2018.06.2856 PMC6333518 30098888

[37] He MS Chang FL Lin HZ Wu JL Hsieh TC Lee YC. The association between diabetes and age–related macular degeneration among the elderly in Taiwan. Diabetes Care (2018) 41(10):2202–11. doi: 10.2337/dc18-0707 30061321

[38] Ulańczyk Z Sobuś A Łuczkowska K Grabowicz A Mozolewska–Piotrowska K Safranow K. Associations of microRNAs, angiogenesis–regulating factors and CFH Y402H polymorphism–an attempt to search for systemic biomarkers in age–related macular degeneration. Int J Mol Sci (2019) 20(22):5750. doi: 10.3390/ijms20225750 31731799 PMC6887747

[39] Hoh RJKZYCYTSSHKHPSMMTMBBLFOB. Complement factor h polymorphism in age–related macular degeneration. Sci (1979). (2005) 308(385):385–9. doi: 10.1126/science.1109557 PMC1512523 15761122

[40] Fisher SA Abecasis GR Yashar BM Zareparsi S Swaroop A Iyengar SK. Meta–analysis of genome scans of age–related macular degeneration. Hum Mol Genet (2005) 14(15):2257–64. doi: 10.1093/hmg/ddi230 15987700

[41] Lukiw WJ Surjyadipta B Dua P Alexandrov PN. Common micro RNAs (miRNAs) target complement factor h (CFH) regulation in alzheimer’s disease (AD) and in age related macular degeneration (AMD). Int J Biochem Mol Biol (2012) 3(1):105–16. doi: 10.1007/s12035-012-8234-4 PMC3325769 22509485

[42] Maller J George S Purcell S Fagerness J Altshuler D Daly MJ. Common variation in three genes, including a noncoding variant in CFH, strongly influences risk of age–related macular degeneration. Nat Genet (2006) 38(9):1055–9. doi: 10.1038/ng1873 16936732

[43] Bazzoni F Rossato M Fabbri M Gaudiosi D Mirolo M Mori L. Induction and regulatory function of miR–9 in human monocytes and neutrophils exposed to proinflammatory signals. Proc Natl Acad Sci USA (2009) 106(13):5282–7. doi: 10.1073/pnas.0810909106 PMC2664036 19289835

[44] Lukiw WJ Pogue AI. Induction of specific microRNA (miRNA) species by ROS–generating metal sulfates in primary human brain cells. J Inorg Biochem (2007) 101(9 SPEC. ISS.):1265-9. doi: 10.1016/j.jinorgbio.2007.06.004 PMC2080079 17629564

[45] Zhao JJ Sun DG Wang J Liu SR Zhang CY Zhu MX. Retinoic acid down–regulates microRNAs to induce abnormal development of spinal cord in spina bifida rat model. Child’s Nervous System. (2008) 24(4):485–92. doi: 10.1007/s00381-007-0520-5 17962954

[46] Krishnan Kutty R Samuel W Jaworski C Duncan T Nagineni CN Raghavachari N. MicroRNA expression in human retinal pigment epithelial (ARPE–19) cells: Increased expression of microRNA–9 by n– (4–hydroxyphenyl) retinamide. Mol Vis (2010) 16:1475–86. PMC2925906 20806079

[47] Moffett HF Novina CD. A small RNA makes a bic difference. Genome Biol (2007) 8:221. doi: 10.1186/gb-2007-8-7-221 17666120 PMC2323214

[48] Rodriguez A Vigorito E Clare S v. WM Couttet P Soond DR. Requirement of bic/microRNA–155 for normal immune function. Sci (1979). (2007) 316(5824):608–11. doi: 10.1126/science.1139253 PMC2610435 17463290

[49] Zhou Q Xiao X Wang C Zhang X Li F Zhou Y. Decreased microRNA–155 expression in ocular behcet’s disease but not in vogt koyanagi harada syndrome. Invest Ophthalmol Vis Sci (2012) 53(9):5665–74. doi: 10.1167/iovs.12-9832 22815348

[50] Bhaumik D Scott GK Schokrpur S Patil CK v. OA Rodier F. MicroRNAs miR–146a/b negatively modulates the senescence–associated inflammatory mediators IL–6 and IL–8. Aging. (2009) 1(4):402–11. doi: 10.18632/aging.100042 PMC2818025 20148189

[51] Beatty S Koh HH Phil M Henson D Boulton M. The role of oxidative stress in the pathogenesis of age–related macular degeneration. Surv Ophthalmol (2000) 45(2):115–34. doi: 10.1016/s0039-6257(00)00140-5 11033038

[52] Lin H Qian J Castillo AC Long B Keyes KT Chen G. Effect of miR–23 on oxidant–induced injury in human retinal pigment epithelial cells. Invest Ophthalmol Vis Sci (2011) 52(9):898–909. doi: 10.1002/stem.1068 21693609

[53] Marion S Hoffmann E Holzer D le Clainche C Martin M Sachse M. Ezrin promotes actin assembly at the phagosome membrane and regulates phago–lysosomal fusion. Traffic. (2011) 12(4):421–37. doi: 10.1111/j.1600-0854.2011.01158.x 21210911

[54] Shalom–Feuerstein R Serror L de la Forest Divonne S Petit I Aberdam E Camargo L. Pluripotent stem cell model reveals essential roles for miR–450b–5p and miR–184 in embryonic corneal lineage specification. Stem Cells (2012) 30(5). 10.1002/stem.1068 22367714

[55] Hinton DR He S Lopez PF. Apoptosis in surgically excised choroidal neovascular membranes in age– related macular degeneration. Arch Ophthalmol (1998) 116(2):203–9. doi: 10.1001/archopht.116.2.203 9488273

[56] Dunaief JL Dentchev T Ying GS Milam AH. The role of apoptosis in age–related macular degeneration. Arch Ophthalmol (2002) 120(11):1435–42. doi: 10.1001/archopht.120.11.1435 12427055

[57] Murad N Kokkinaki M Gunawardena N Gunawan MS Hathout Y Janczura KJ. MiR–184 regulates ezrin, LAMP–1 expression, affects phagocytosis in human retinal pigment epithelium and is down–regulated in age–related macular degeneration. FEBS J (2014) 281(23):5251–64. doi: 10.1111/febs.13066 25251993

[58] Huynh KK Eskelinen EL Scott CC Malevanets A Saftig P Grinstein S. LAMP proteins are required for fusion of lysosomes with phagosomes. EMBO J (2007) 26(2):313–24. doi: 10.1038/sj.emboj.7601511 PMC1783450 17245426

[59] Wang S Olson EN. AngiomiRs–key regulators of angiogenesis. Curr Opin Genet Dev (2009) 19:205–11. doi: 10.1016/j.gde.2009.04.002 PMC2696563 19446450

[60] Shen JK Yang X Xie B Chen Y Swaim M Hackett SF. MicroRNAs regulate ocular neovascularization. Mol Ther (2008) 16(7):1208–16. doi: 10.1038/mt.2008.104 PMC3033219 18500251

[61] Chen Y Gorski DH. Regulation of angiogenesis through a microRNA (miR–130a) that down–regulates antiangiogenic homeobox genes GAX and HOXA5. Blood (2008) 111(3):1217–26. 10.1182/blood-2007-07-104133 PMC2214763 17957028

[62] Sabatel C Malvaux L Bovy N Deroanne C Lambert V Gonzalez MLA. MicroRNA–21 exhibits antiangiogenic function by targeting RhoB expression in endothelial cells. PLoS One (2011) 6(2):e16979. doi: 10.1371/journal.pone.0016979 21347332 PMC3037403

[63] Grossniklaus HE Kang SJ Berglin L. Animal models of choroidal and retinal neovascularization. Prog Retinal Eye Res (2010) 29:500–19. doi: 10.1016/j.preteyeres.2010.05.003 PMC2962694 20488255

[64] Kuehbacher A Urbich C Dimmeler S. Targeting microRNA expression to regulate angiogenesis. Trends Pharmacol Sci (2008) 29(1):12–5. doi: 10.1016/j.tips.2007.10.014 18068232

[65] Bajan S Hutvagner G. RNA–Based therapeutics: From antisense oligonucleotides to miRNAs. Cells. (2020) 9(1):137. doi: 10.3390/cells9010137 31936122 PMC7016530

[66] Romano GL Platania CBM Drago F Salomone S Ragusa M Barbagallo C. Retinal and circulating miRNAs in age–related macular degeneration: An in vivo animal and human study. Front Pharmacol (2017) 8:168. doi: 10.3389/fphar.2017.00168 28424619 PMC5371655

[67] Hauswirth WW Aleman TS Kaushal S v. CA SB S Wang L. Treatment of leber congenital amaurosis due to RPE65 mutations by ocular subretinal injection of adeno–associated virus gene vector: Short–term results of a phase I trial. Hum Gene Ther (2008) 19(10):979–90. doi: 10.1089/hum.2008.107 PMC2940541 18774912

[68] Bainbridge JWB Smith AJ Barker SS Robbie S Henderson R Balaggan K. Effect of gene therapy on visual function in leber’s congenital amaurosis. New Engl J Med (2008) 358(21):2231–9. doi: 10.1056/NEJMoa0802268. 18441371

[69] Hyttinen JMT Blasiak J Felszeghy S Kaarniranta K. MicroRNAs in the regulation of autophagy and their possible use in age–related macular degeneration therapy. Ageing Res Rev (2021) 67:101260. doi: 10.1016/j.arr.2021.101260 33516915

[70] Zhou Q Gallagher R Ufret–Vincenty R Li X Olson EN Wang S. Regulation of angiogenesis and choroidal neovascularization by members of microRNA–23∼27∼24 clusters. Proc Natl Acad Sci USA (2011) 108(20):8287–92. 10.1073/pnas.1105254108 PMC3100947 21536891

[71] Roblain Q Louis T Yip C Baudin L Struman I Caolo V. Intravitreal injection of anti–miRs against miR–142–3p reduces angiogenesis and microglia activation in a mouse model of laser–induced choroidal neovascularization. Aging. (2021) 13(9):12359–77. doi: 10.18632/aging.203035 PMC8148470 33952723

[72] Bak RO Mikkelsen JG. miRNA sponges: Soaking up miRNAs for regulation of gene expression. Wiley Interdiscip Reviews: RNA. (2014) 5(3):317–33. doi: 10.1002/wrna.1213 24375960

[73] Bak RO Hollensen AK Primo MN Sørensen CD Mikkelsen JG. Potent microRNA suppression by RNA pol II–transcribed “Tough decoy” inhibitors. RNA. (2013) 19(2):280–93. doi: 10.1261/rna.034850.112 PMC3543086 23249752

[74] Bak RO Hollensen AK Mikkelsen JG. Managing microRNAs with vector–encoded decoy–type inhibitors. Mol Ther (2013) 21(8):1478–85. doi: 10.1038/mt.2013.113 PMC3734669 23752312

